# Monitoring Spike Potential and Abrupt Impedance Rise with Concomitant Temperature/Contact Force Change for Timely Detection of the Occurrence of “Silent” or “Nonaudible” Steam Pop

**DOI:** 10.1155/2023/8873404

**Published:** 2023-04-05

**Authors:** Chengye Di, Qun Wang, Yanxi Wu, Longyu Li, Wenhua Lin

**Affiliations:** ^1^Cardiac Electrophysiology Unit, First Department of Cardiology, TEDA International Cardiovascular Hospital, Tianjin, China; ^2^College of Clinical Cardiology, Tianjin Medical University, Tianjin, China; ^3^Cardiovascular Institute, Tianjin University, Tianjin, China

## Abstract

**Aim:**

Steam pop (SP) during radiofrequency catheter ablation (RFCA) for pulmonary vein isolation (PVI) may cause cardiac perforation, which may require drainage and emergent thoracotomy or even lead to death. Data investigating the timely detection of the occurrence of “silent” or “nonaudible” SP events are limited.

**Methods and Results:**

A total of 516 consecutive atrial fibrillation (AF) patients who underwent index PVI were included in this retrospective observational study. The duration, power, impedance, temperature, and contact force (CF) of RFCA were continually monitored and recorded throughout the procedure. A total of 15 (2.9%) audible SP events occurred in 14 patients; 2 of the patients developed pericardial tamponade, 1 patient underwent drainage, and 1 patient underwent emergent thoracotomy. The time from RFCA initiation to the occurrence of audible SP was 19.4 ± 6.9 s. Abrupt temperature change occurred in 13 (86.7%) of the 15 SP events, of which 8 (53.3%) exhibited an abrupt temperature rise of 2.3 ± 1.0°C, 5 (33.3%) exhibited an abrupt temperature drop of 2.3 ± 1.3°C, and 2 (13.3%) exhibited no discernible temperature change.

**Conclusions:**

In conclusion, simultaneously recorded spike potentials and abrupt impedance rise with concomitant temperature and/or CF change could be a feasible method for the timely detection of the occurrence of audible, “silent,” or “nonaudible” SP events, particularly in regions where the risk of perforation may be of concern.

## 1. Introduction

Pulmonary vein isolation (PVI) utilized radiofrequency catheter ablation (RFCA) has emerged as an effective ablation strategy for the treatment of patients with atrial fibrillation (AF) [1]. Achieving durable PVI is necessary for the efficacy of RFCA for AF. The formation of lesions caused by RFCA current depends on several parameters, such as the duration, temperature, impedance, and contract force (CF). Radiofrequency current energy creates myocardial lesions by causing the resistive heating of the myocardial tissue, followed by heat conduction to the adjacent tissue [2, 3]. Permanent thermal injury occurs in regions where the temperature reaches 50°C and above. If the temperature of the tissue exceeds 100°C, steam explosions can occur, which may be audible as “steam pop (SP)” events [3]. SP events may cause the potential consequences of atrial hematoma, ventricular septal defect, thrombus formation, embolism, laceration of the coronary artery, cardiac perforation, and even dramatic complications that require emergent thoracotomy [4–9]. Restricting the parameters of RFCA has been advocated as a method to prevent SP events [5, 10, 11]. Data investigating the timely detection of the occurrence of “silent” or “nonaudible” SP events are limited. This study sought to assess the feasibility of monitoring the recorded spike potential and abrupt rise in impedance with concomitant temperature and/or CF change as an indicator for timely detection of the occurrence of “silent” or “nonaudible” SP events, particularly in regions where the risk of perforation may be of concern.

## 2. Methods

### 2.1. Study Setting

Of 516 consecutive patients undergoing index PVI for paroxysmal or persistent AF between January 2017 and November 2020, a total of 15 (2.9%) audible SP events were included in this retrospective observational study. Patients with a left atrial (LA) or left atrial appendage (LAA) thrombus, valvular heart disease, prior valve replacements, a history of PVI, or LA diameter (anterior-posterior) of >50 mm on transthoracic echocardiography (TTE) were excluded. The demographic and clinical data were collected prior to the procedure. This study complied with the Declaration of Helsinki.

#### 2.1.1. Preprocedural Management

All antiarrhythmic drugs (AADs) were discontinued at least 5 half-lives before the procedure. All patients underwent a preprocedural computed tomographic (CT) scan to assess the detailed LA and pulmonary vein (PV) anatomy. Transthoracic echocardiography (TTE) was performed within 14 days before the RFCA procedure, enabling the assessment of the LA and left ventricular (LV) function and intracavity dimensions. Transesophageal echocardiography (TEE) was performed 2 days before the RFCA procedure to exclude the possible presence of thrombi in the LA and/or the LAA.

### 2.2. RFCA Power Settings and PVI

Patients were treated with conscious analgesia with fentanyl citrate during the RFCA procedure. A decapolar catheter was positioned within the coronary sinus. The circumferential decapolar mapping catheter (Lasso, Biosense Webster, Diamond Bar, CA, USA) was sequentially positioned in each PV antrum to obtain baseline PV potential (PVP) information. All patients underwent PVI using a 3.5 mm externally irrigated-tip ablation catheter (SmartTouch, D curve or F curve, Biosense Webster, Diamond Bar, CA, USA). After fast anatomic map of the LA and PV, we use posterior-anterior and left-lateral projection to make “zero” on the ablation catheter while looking at the live CF graph. RFCA for PVI was performed at a power control mode of 35 W at the roof and posterior wall and 40 W at the anterior wall with irrigation mode at a flow rate of 17 mL/min. Ablation was guided by ablation index (AI) target values for each lesion as follows: 500–550 for anterior/roof segments and 250–350 for posterior/inferior segments of the PV. The RFCA parameters of duration, power, impedance, temperature, and CF were continuously monitored and recorded throughout the procedure, and the data were made readily available for retrieval and analysis offline by the operator. RFCA was terminated immediately if an audible SP occurred. Spike potential was defined as a high-frequency and amplitude potential recorded immediately during the occurrence of an audible SP. Steerable sheath (MobiCath, Biosense Webster, Diamond Bar, CA, USA) was advanced alternately only when the nonsteerable sheath (8.5 F SL1, St. Jude Medical, St. Paul, MN, USA) could not advance the ablation catheter to the PV antrum. PVI was defined by the elimination or dissociation of PVPs recorded on the Lasso catheter.

### 2.3. Audible SP and RFCA Parameter Measurements

The impedance drop was defined as the baseline impedance minus the minimum impedance observed during RFCA application, either with or without SP. An audible SP was defined as an audible sound both heard by the operator and the patient during RFCA. The time from the initiation of RFCA to the SP and AI at the time of the SP were recorded. The location of the audible SP occurring during RFCA was marked in a map. The changes in the values of the RFCA parameters were measured as the difference between the parameter values after the SP and those just before the SP. No further RFCA application was applied until perforation is excluded in the vicinity of the audible SP. A control lesion was chosen at the nearest RFCA location preceding the SP. If no suitable control lesion occurred before a lesion with a SP, the closest following RFCA lesion fulfilling the criteria was selected. Patients underwent immediate TTE after the occurrence of an audible SP, at the end of the procedure, and on the day after the RFCA procedure.

### 2.4. Statistical Analysis

Normal distribution was verified by the Kolmogorov–Smirnov test, and data are presented as mean ± standard deviation (SD). Categorical variables are presented as numbers and/or percentages in parentheses. When 2 groups were compared, either Student's test or the Mann–Whitney *U* test was used according to the distribution of the data. In this analysis, 2-tailed tests were performed, and *P* values <0.05 were considered statistically significant. Statistical analysis was performed with SPSS software (SPSS Inc., Version 22).

## 3. Results

### 3.1. Patient Characteristics

The baseline clinical characteristics of the patients in the study population are summarized in Table 1. The mean age was 59 years old (40.0% male). The CHA_2_DS_2_-VASc score was 3.1 ± 1.1, and the HAS-BLED score was 2.7 ± 0.7. Nine (60.0%) of the 15 patients exhibited paroxysmal AF, 4 (26.7%) had hypertension, 5 (33.3%) had diabetes mellitus, and 4 had coronary heart disease. The LA diameter was 43.1 ± 3.2 mm, the left ventricular end-diastolic dimension was 49.0 ± 3.4 mm, and the left ventricular ejection fraction was 57.6 ± 8.5%. Steerable sheath was used in 1 of the 15 patients (6.7%).

### 3.2. Localizations of Audible SPs

**Figure 1:**
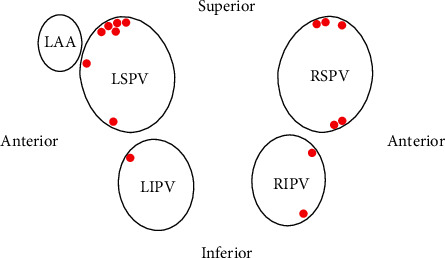
The large circles indicate the left superior pulmonary vein (LSPV), left inferior pulmonary vein (LIPV), right superior pulmonary vein (RSPV), right inferior pulmonary vein (RIPV), and left atrial appendage (LAA). The distribution of steam pop (SP) locations during radiofrequency catheter ablation (RFCA) for PVI is marked in red points in the posterior-anterior view of the left atrium.

**Figure 2:**
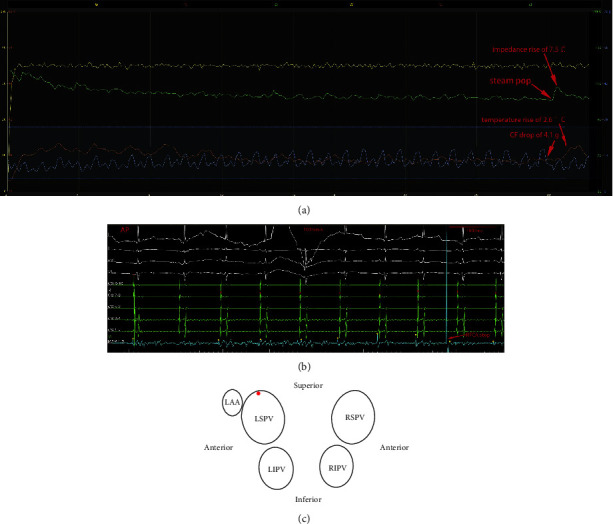
(a) RFCA graph showing the course of the parameters during RFCA. The time from the initiation of RFCA to the occurrence of audible SP was 30.2 s. The occurrence of a SP was associated with a preceding impedance drop of 17.6 Ω. The sudden impedance rise after 30.2 s of RFCA coincided with a SP. Immediately after the SP, an abrupt impedance rise of 7.5 Ω, temperature rise of 2.6°C, and CF drop of 4.1 g were recorded. (b) A spike potential was not recorded immediately during the occurrence of SP. (c) The location of the SP is at the anterior-superior wall of the LSPV.

**Figure 3:**
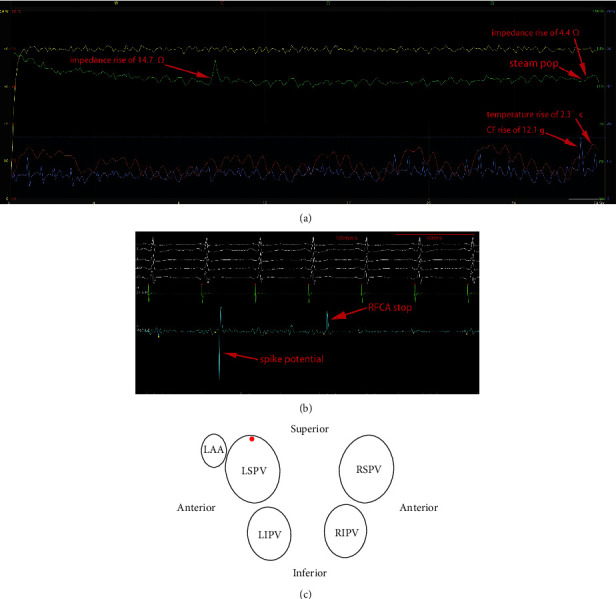
(a) The time from the initiation of RFCA to the occurrence of audible SP was 27.3 s. The occurrence of SP was associated with a preceding impedance drop of 18.5 Ω. The sudden impedance rise after 27.3 s of RFCA coincided with a SP. Immediately after the SP, an abrupt impedance rise of 4.4 Ω, temperature rise of 2.3°C, and CF rise of 12.1 g were recorded. (b) A spike potential without consequent PAC was recorded immediately during the occurrence of SP. Of note, this RFCA application had 1 previous significant impedance rise of 14.7 Ω at 9.4 s but without concomitant temperature and CF change. (c) The location of the SP is at the roof wall of the LSPV.

**Figure 4:**
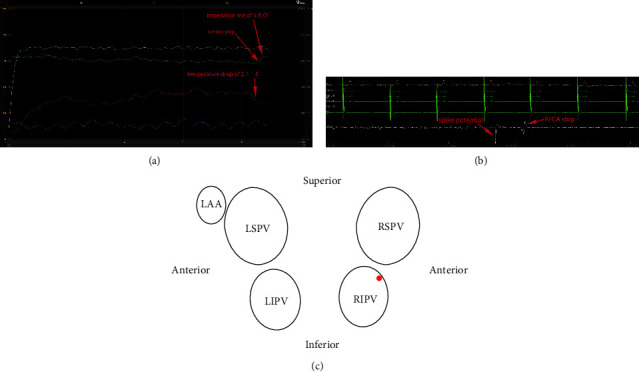
(a) The time from the initiation of RFCA to the occurrence of audible SP was 8.6 s. The sudden impedance rise after 8.9 s of RFCA coincided with a SP. Immediately after the SP, an abrupt impedance rise of 5.8 Ω, temperature drop of 2.1°C, and CF without discernible change were recorded. (b) A spike potential without consequent PAC was recorded immediately during the occurrence of SP. (c) The location of the SP is at the anterior-superior wall of the RIPV.

The locations of the 15 SPs varied and were widely distributed to the 4 PVs (Figure 1). Five (33.3%) SPs occurred at the anterior-superior (Figure 2) or roof wall (Figure 3), 1 (6.7%) SP occurred at the anterior wall, and 1 (6.7%) SP occurred at the anterior-inferior wall of the left superior pulmonary vein (LSPV). One (6.7%) SP occurred at the anterior-superior wall of the left inferior pulmonary vein (LIPV). One (6.7%) SP occurred at the anterior-superior wall (Figure 4), and 1 (6.7%) SP occurred at the anterior-inferior wall of the right inferior pulmonary vein (RIPV). Two (13.3%) SPs occurred at the anterior-inferior wall, and 3 (20%) SPs occurred at the anterior-superior wall or posterior-superior wall of the right superior pulmonary vein (RSPV).

### 3.3. Incidence of Audible SP and Results

Of 516 consecutive patients undergoing index PVI for AF, a total of 15 (2.9%) audible SPs in 14 patients were recorded in this study. Two of the patients developed pericardial tamponade, 1 patient underwent drainage, 1 patient underwent emergent thoracotomy (Figure 3), and no patients died. The occurrence of SPs may have changed RFCA settings used by the operator during subsequent RFCA applications. Therefore, the RFCA lesions generated immediately before lesions with SPs were preferentially selected as controls. However, 2 of 15 (13.3%) control lesions were generated after the occurrence of RFCA lesions with SPs because suitable control lesions occurring before the lesions with SPs were lacking.

### 3.4. Analysis of RFCA Parameters before the Occurrence of Audible SP

The time from the initiation of RFCA to the occurrence of audible SP was 19.4 ± 6.9 s. The AI prior to the occurrence of audible SP was 392.4 ± 87.1. The minimum temperature was significantly higher in the audible SP group than in the control group (34.5 ± 1.7 vs. 31.5 ± 2.1°C, *P* < 0.001). The maximum impedance preceding SP was higher in the audible SP group than in the control group (127.2 ± 11.9 vs. 119.8 ± 6.9 Ω, *P* < 0.001). The impedance drop preceding SP was significantly higher in the audible SP group than in the control group (20.8 ± 9.5 vs. 8.2 ± 4.1 Ω, *P* < 0.001). In the audible SP group, 2 (13.3%) of the 15 SPs were associated with an impedance drop of <10 ohm, 2 (13.3%) of the 15 SPs were associated with an impedance drop between 10 and 15 Ω, 5 (33.3%) of the 15 SPs were associated with an impedance drop between 15 and 20 Ω, and 6 (40%) of the 15 SPs were associated with an impedance drop of >20 Ω. The maximum CF, minimum CF, and mean CF preceding SP were significantly higher in the audible SP group than in the control group (17.7 ± 4.1 vs. 13.6 ± 3.9 g, *P*=0.001; 5.6 ± 3.6 vs. 3.6 ± 3.6 g, *P*=0.009; and 11.3 ± 2.6 vs. 7.7 ± 1.6 g, *P* < 0.001, respectively) (Table 2).

### 3.5. Analysis of RFCA Parameters prior to and after the Occurrence of Audible SP

The real-time impedance prior to the occurrence of audible SP was 110.9 ± 9.7 Ω; immediately after the occurrence of audible SP, 14 (93.3%) of the 15 SPs exhibited an abrupt impedance rise of 11.9 ± 9.7 Ω, and only 1 SP without a discernible impedance change was observed. The real-time CF prior to the occurrence of audible SP was 13.6 ± 4.2 g; an immediate change in CF occurred in 12 (80%) of the 15 SPs; of the 15 total SPs, 3 (20%) exhibited an abrupt CF rise of 6.7 ± 1.0 g, 9 (60%) exhibited an abrupt CF drop of 8.4 ± 5.5 g, and 3 (20%) exhibited no discernible change in CF. Ten (66.7%) spike potentials were recorded immediately during the occurrence of audible SP (Table 3).

### 3.6. Data regarding Prospective Evaluation

**Figure 5:**
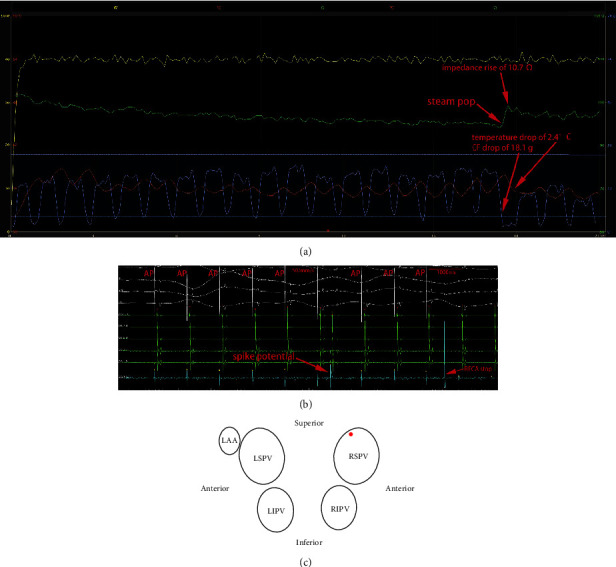
(a) In the prospective RFCA procedures, simultaneously recorded spike potential and abrupt rise in impedance with concomitant temperature and CF drop could timely detect the occurrence of “silent” SP. The time from the initiation of RFCA to the occurrence of “nonaudible” SP was 17.4 s. The sudden impedance rise after 17.4 s of RFCA coincided with a SP. After the “nonaudible” SP, an abrupt impedance rise of 10.7 Ω, temperature drop of 2.4°C, and CF drop of 18.1 g were recorded. The RFCA was terminated approximately 3.5 s later. (b) A spike potential with a consequent PAC was recorded immediately during the occurrence of SP. (c) The location of the SP is at the posterior-superior wall of the RSPV. This patient subsequently developed pericardial tamponade and underwent drainage. The patient received a dual-chamber pacemaker for sick sinus syndrome 2 years ago. AP: atrial pacing.

In 217 prospective RFCA procedures, the simultaneously recorded spike potential and abrupt rise in impedance with concomitant temperature and/or CF change could timely detect the occurrence of “silent” or “nonaudible” SPs in 2 cases. In 1 case, after the “nonaudible” SP, an abrupt impedance rise of 10.7 Ω, temperature drop of 2.4°C, and CF drop of 18.1 g were recorded. A spike potential with a consequent PAC was recorded immediately during the occurrence of SP. The RFCA was terminated approximately 3.5 s later (Figure 5). This patient developed pericardial tamponade and underwent drainage.

## 4. Discussion

This retrospective observational study provides new data about SP occurring during RFCA for PVI. First, approximately 2.9% of all RFCA applications resulted in the occurrence of an audible SP and a consequent pericardial tamponade occurrence rate of 13.3%. Second, the time from the initiation of RFCA to audible SP occurrence was 19.7 ± 7.3 s, the AI prior to audible SP occurrence was 392.4 ± 87.1, and the AI could not be used to predict the occurrence of SP. Third, the simultaneously recorded spike potential and abrupt rise in impedance with concomitant temperature and/or CF change could be a feasible method to timely detect the occurrence of audible, “silent,” or “nonaudible” SPs, particularly in regions where the risk of perforation may be of concern.

The decrease in impedance during RFCA application is assumed to be the result of ion mobility [12, 13]. Several studies have reported that the magnitude of the impedance drop predicts acute PVI [14–16]. An impedance drop of >15–18 ohm was found to be associated with an increased risk of SP occurrence [17, 18]. Nguyen et al. reported that most occurrences of SP were related to a rapid drop in impedance within the first 5 s of RFCA [5]. However, the precise range of the drop in impedance or the timing of when SP occurs is relatively unknown [19]. There is an overlap between the occurrence of the drop in impedance and the occurrence of audible SP, and knowledge regarding the prognostic value of drops in impedance as a parameter for assessing the safety of RFCA is very limited [9, 11]. In the present study, 11 (73.3%) of the 15 audible SPs were associated with a preceding drop in impedance of >15 ohm, indicating the limited prognostic value of drops in impedance as an indicator of safe RFCA (Figure 4).

The duration of RFCA application in the audible SP group was shorter than that in the control group (19.4 ± 6.9 vs. 31.7 ± 5.0 s, *P* < 0.001), and the AI in the audible SP group was also smaller than that in the control group (392.4 ± 87.1 vs. 485.0 ± 28.6, *P*=0.001). This comparison was influenced by the RFCA duration when a SP occurred and RFCA delivery was stopped. The duration, power, impedance, temperature, and CF are all effective and important factors for RFCA lesion formation [2]. Recent studies have shown that AI is a novel guidance parameter for predicting durable PVI during ablation [20, 21]. However, the AI does not account for the effects of baseline impedance and drops in impedance; thus, although the AI is useful to deliver adequate lesion formation, it is inadequate to forecast SP occurrence (Figure 4) [21–23].

The power and duration of RFCA are clinically controllable parameters that influence lesion formation and SP occurrence. Likewise, electrode-tissue coverage seems to have a significant influence on lesion formation, as it affects the amount of RF current that drives the ablation process [24]. Animal model studies have identified a significant association between drops in impedance, lesion dimension, observed SP occurrence, and crater formation, which is associated with larger drop in impedance, but no specific cutoff could be defined [25, 26]. Mori et al. demonstrated that the CF values of ablations resulting in audible SPs were not higher than those without SPs [21]. Cooper et al. found that the mean catheter tip temperature at the time of steam explosions identified by intracardiac echocardiography (ICE) was 43.6°C. They recommended maintaining a catheter tip temperature of <40°C to prevent SPs [11]. In this study, the mean catheter tip temperature was 39°C at the time of SPs, and there was no significant difference in the maximum catheter tip temperature in lesions with and without SPs. Furthermore, SPs were observed at catheter tip temperatures as low as 34°C [17]. The minimum temperature was significantly higher in the audible SP group than that in the control group (34.5 ± 1.7 vs. 31.5 ± 2.1°C, *P* < 0.001). The results of the present study are consistent with the results of previous reports, and SPs occurred at a minimum RFCA time of 8.9 s (Figure 4). Shortening the RFCA duration and using lower power and CF are feasible and reliable methods to prevent the occurrence of SP.

As gas is almost an electrical insulation during SP, its presence within the ablated tissue will result in an increase in the measured total impedance, and thus an abrupt rise in impedance is often observed during a SP [27]. We hypothesized that the SP may lead to temporary displacement of the catheter tip to an adjacent area. In SPs with temperature and/or CF drop-off, the force of the SP may displace the catheter tip toward the LA cavity, and the catheter tip may fail to immediately contact the LA wall after the onset of the SP, which leads to the temperature and/or CF value dropping (Figure 5). In the SPs exhibiting temperature and/or CF increase, the force of the SP may not displace the catheter tip; thus, the catheter tip can sense the force of boiling and the explosions of the SP, which leads to a sudden temperature and/or CF increase (Figure 3). A nondiscernible change in the temperature and/or CF is a manifestation of the immediate rebounding of the catheter tip against the LA wall after SP; thus, the initial release of force after SP will be followed by the recovery of the temperature and/or CF.

Silent SP can be observed by ICE recording; however, audible sound and sudden changes in impedance and temperature are not always present when visible explosions occur during ICE [28, 29]. Ganapathy et al. showed that CF recovery could be used to predict the absence of cardiac perforation during SPs [30]. However, in fact, the so-called “silent” or “nonaudible” SP was sometimes just unheard by the operator. Thus, a method that could alert operators to timely detect the occurrence of “silent” or “nonaudible” SP in a clinical setting would have great clinical value. Based on the recorded spike potential and RFCA parameter changes during these 15 audible SPs, we speculate that simultaneously recorded spike potential and abrupt rise in impedance, temperature, and (or) CF change could be used (1) to predict the occurrence of SP with an accuracy of 97.8%; (2) to predict the occurrence of SP with an accuracy of 99.9%; (3) to predict the occurrence of SP with an accuracy of 99.7%; and (4) to predict the occurrence of SP with an accuracy of 99.9%. We presumed that, in fact, the so-called “silent” or “nonaudible” SP could be timely detected based on the abovementioned parameters.

### 4.1. Study Limitations

There are some limitations in this study. Firstly, we assessed only 15 SPs in this study. However, SPs are very rare in the clinical setting, so it may be difficult to obtain a considerable number of cases. However, detailed RFCA parameters were evaluated, and further work should be performed in the future. Secondly, only audible SPs were included, and these may reflect the more severe consequences of the range of steam explosions that are possible. Silent or small SPs without sudden changes in RFCA parameters may be observed by ICE imaging; however, we did not use ICE imaging to assess SPs in this study. Thirdly, individual clinical cases varied depending on patient habitus and underlying pathological substrates. Further studies are warranted to validate the clinical significance of these findings.

## 5. Conclusions

Based on the recorded spike potential and RFCA parameter changes observed during the 15 audible SPs, we speculate that either with the audible or “nonaudible” SPs, simultaneously recorded spike potential and abrupt rise in impedance with concomitant temperature and/or CF change could be used to timely detect the occurrence of SP. This is a feasible method to enable the timely detection of the occurrence of either audible, “silent,” or “nonaudible” SPs, particularly in regions where the risk of perforation may be of concern and even present a serious complication.

## Figures and Tables

**Table 1:** Baseline clinical characteristics of the study population.

	*N* = 15
Age (years)	59 ± 13
Male sex (%)	6/15 (40.0%)
Hypertension (%)	4/15 (26.7%)
DM (%)	5/15 (33.3%)
CHD (%)	4/15 (26.7%)
Stroke (%)	1/15 (6.7%)
Paroxysmal AF patients (%)	9/15 (60.0%)
AF history (months)	15.5 ± 19.8^*∗*^
CHA_2_DS_2_-VASc score	3.1 ± 1.1^*∗*^
HAS-BLED score	2.7 ± 0.7^*∗*^
BMI (kg/m^2^)	28.0 ± 2.9
K (mmol/L)	4.0 ± 0.4
Cr (*μ*mol/L)	70.5 ± 8.3
UA (*μ*mol/L)	355.5 ± 46.1
Glu (mmol/L)	5.5 ± 1.0^*∗*^
LA (mm)	42.8 ± 3.2
LVEDD (mm)	49.0 ± 3.4
LVEF (%)	57.6 ± 8.5

Values are given as mean ± SD. ^*∗*^Nonnormally distributed data. BMI = body mass index; LA = left atrium; DM = diabetes mellitus; CHD = coronary heart disease; AF = atrial fibrillation; LVEDD = left ventricular end-diastolic dimension; LVEF = left ventricular ejection fraction.

**Table 2:** Comparison of RFCA parameters between audible steam pop group and control group during pulmonary vein isolation (power: 35–40 W).

	Audible steam pop group (*N* = 15)	Control group (*N* = 15)	*t*/*Z*/*χ*2 value	*P* value
Time from RFCA initiation to occurrence of audible steam pop (s)	19.4 ± 6.9	31.7 ± 5.0^#^	5.610	<0.001
AI prior to audible steam pop	392.4 ± 87.1	485.0 ± 28.6^#^	3.914	<0.001
Maximum temperature preceding steam pop (°C)	38.8 ± 2.0	38.0 ± 2.4	1.022	0.315
Minimum temperature preceding steam pop (°C)	34.5 ± 1.7	31.5 ± 2.1	4.273	<0.001
Mean temperature preceding steam pop (°C)	36.2 ± 1.6	35.7 ± 2.3^*∗*^	1.143	0.267
Maximum impedance preceding steam pop (Ω)	127.2 ± 11.9	119.8 ± 6.9	2.090	0.046
Minimum impedance preceding steam pop (Ω)	106.4 ± 8.9	111.6 ± 6.1^*∗*^	1.663	0.090
Mean impedance preceding steam pop (Ω)	117.0 ± 10.3	115.2 ± 5.7	0.590	0.561
Impedance drop preceding steam pop (Ω)	20.8 ± 9.5	8.2 ± 4.1	4.753	<0.001
Maximum CF preceding steam pop (g)	17.7 ± 4.1	13.6 ± 3.9^*∗*^	3.288	<0.001
Minimum CF preceding steam pop (g)	5.6 ± 3.6	3.6 ± 3.6^*∗*^	2.598	0.009
Mean CF preceding steam pop (g)	11.3 ± 2.6	7.7 ± 1.6^*∗*^	3.517	<0.001

Values are given as mean ± SD. ^#^RF parameters without steam pop. ^*∗*^Nonnormally distributed data. RFCA = radiofrequency catheter ablation; CF = contact force; AI = ablation index.

**Table 3:** RFCA parameters prior to and after the occurrence of audible steam pop during pulmonary vein isolation (power: 35–40 W).

	Audible steam pop group (*N* = 15)
Real-time temperature prior to audible steam pop (°C)	37.1 ± 1.7
Abrupt temperature change after audible steam pop	
Temperature rise (°C, *n*)	2.3 ± 1.0, 8
Temperature drop (°C, *n*)	2.3 ± 1.3^*∗*^, 5
Temperature without discernible change (*n*)	2
Real-time impedance prior to audible steam pop (Ω)	110.9 ± 9.7
Abrupt impedance change after audible steam pop	
Impedance rise (Ω, *n*)	11.8 ± 9.2^*∗*^, 14
Impedance drop (Ω, *n*)	0, 0
Impedance without discernible change (*n*)	1
Real-time CF prior to audible steam pop (g)	13.6 ± 4.2
Abrupt CF change after audible steam pop	
CF rise (g, *n*)	6.7 ± 1.0^*∗*^, 3
CF drop (g, *n*)	8.4 ± 5.5, 9
CF without discernible change (*n*)	3
Spike potential recorded during audible steam pop (%)	10 (66.7%)
Pericardial tamponade occurred after audible steam pop (%)	2 (13.3%)

Values are given as mean ± SD. Spike potential was defined as a high-frequency and amplitude potential recorded during the audible steam pop. ^*∗*^Nonnormally distributed data. Data in the parentheses indicate RF parameters, percentage, and case numbers. RF = radiofrequency; CF = contact force.

## Data Availability

The clinical and procedural data used to support the findings of this study are included within the article.
